# The effects of berberine on depressive symptoms: a systematic review and meta-analysis of preclinical studies

**DOI:** 10.3389/fpsyt.2025.1653929

**Published:** 2025-12-29

**Authors:** Xiaona Li, Mei Lu, Xinkun Wang, Di Wu, Yongmei Chen, Ming Tao, Dengqun Gou, Runyu Yang, Jiamei Zhou

**Affiliations:** 1 Department of Nursing, Affiliated Hospital of Zunyi Medical University, Zunyi, China; 2 Nursing School of Zunyi Medical University, Zunyi, China; 3 Affiliated Hospital of Inner Mongolia Medical University, Hohhot, China; 4 Department of Hematology, West China Hospital, Sichuan University/West China School of Nursing, Sichuan University, Chengdu, China

**Keywords:** depression, berberine, coptisine, meta-analysis, animal mode, systematic review

## Abstract

**Introduction:**

Despite preclinical evidence for berberine's antidepressant potential, its pharmacological effects remain controversial.This study therefore systematically reviews animal research to clarify its mechanisms and support future clinical trials.

**Methods:**

We searched PubMed, Embase, Web of Science, Cochrane Library, and OVID for studies on berberine in depression models up to March 20, 2025. Analysis used STATA 15.0 and Review Manager 5.4, with study quality assessed via SYRCLE's risk of bias tool.

**Results:**

The meta-analysis included 18 studies (338animals). Overall, berberine significantly reduced depression-like behaviors in animal models.Specifically, BBR increased total locomotor activity in the open field test (SMD=2.79, 95% CI: 1.55, 4.02) and time spent in the center zone (SMD=2.49, 95% CI:1.61, 3.37), reduced immobility time in both the forced swim test and tail suspension test (SMD =-4.42, 95% CI:-5.77,-3.07; SMD=-4.46, 95% CI:-6.21, -2.71), increased sucrose intake in the sucrose preference test (SMD = 3.72, 95% CI: 2.37, 5.07), and reduced feeding latency in the novelty-suppressed feeding test (SMD=-5.72, 95% CI:-7.63, -3.82). However, BBR did not significantly alter the number of square crossings (SMD=1.36, 95%CI:-0.07 , 2.79) or rearing frequency (SMD=1.66, 95% CI: -0.29, 3.61) in the open field test. BBR also increased the levels of body weight, brain-derived neurotrophic factor, dopamine, serotonin, and norepinephrine,while reducing the levels of pro-inflammatory cytokines including TNF-α, IL-1β, and IL-6.

**Discussion:**

Preclinical studies suggest that berberine may represent a promising therapeutic agent for the treatment of depressive disorders. Its antidepressant effects appear to be closely associated with the modulation of neurotransmitter levels,reduction of oxidative stress, and inhibition of inflammatory responses.However, methodological limitations may constrain these findings. Larger, more rigorous preclinical studies are needed for confirmation.

**Systematic review registration:**

https://inplasy.com/inplasy-2025-6-0002, identifier INPLASY202560002.

## 1 Introduction

Depressive disorder (DD) is a common psychiatric condition characterized by symptoms such as low mood, cognitive impairment, sleep disturbances, social withdrawal, and reduced motivation, and may lead to self-injurious behavior in severe cases (1). Current epidemiological data indicate a global prevalence of approximately 5% for depressive disorders (1). According to disability-adjusted life year (DALY) estimates, depressive disorders accounted for 1.85% of the total global disease burden and increased by 61.1% between 1990 and 2019 (2). The World Health Organization (WHO) has recognized depression as the primary contributor to worldwide disability (3).

Currently, commonly prescribed pharmacological treatments for depressive disorders include selective serotonin reuptake inhibitors, serotonin-norepinephrine reuptake inhibitors, monoamine oxidase inhibitors, and tricyclic and tetracyclic antidepressants (4). However, these medications typically have a delayed onset of action, and approximately one-third of patients with major depressive disorder show inadequate symptom improvement despite using multiple antidepressants (5). These drugs are linked to various adverse effects, which encompass, but are not restricted to, gastrointestinal issues, liver toxicity, allergic reactions, increased body weight, metabolic abnormalities, sexual dysfunction, and sleep-related disorders (6–8). Carvalho et al. have proposed that the prolonged administration of these novel antidepressants ought to be discouraged in the presence of alternative therapeutic options. (9). Given the limitations of current therapies, there exists a pressing need within the clinical setting for antidepressant therapies that are both safer and more efficacious. In this context, the identification of natural compounds with antidepressant properties from traditional herbal medicine has emerged as a promising direction in medical research.

Berberine (BBR), chemically known as 5,6-dihydro-9,10-dimethoxybenzo[g]-1,3-benzodioxolo[5,6-a]quinolizinium, a quaternary ammonium alkaloid, is a major active component found in several traditional medicinal herbs, including Chinese goldthread (Coptis chinensis), nutgrass (Cyperus rotundus), and rhubarb (Rheum palmatum), and is known to exert a wide range of pharmacological effects (10). As a representative alkaloid among natural products, BBR possesses a multi-target and multi-pathway pharmacological profile. It has been confirmed to exhibit various biological activities, including hypoglycemic (11), lipid-modulating (12), antibacterial (13), anti-inflammatory (14), and anti-tumor effects (15), demonstrating significant clinical translational potential. Notably, the impact of berberine in the neuropsychiatric field has garnered widespread attention in recent years. A growing body of research has confirmed that BBR exhibits significant antidepressant effects, primarily through mechanisms such as modulation of neurotransmitter levels, promotion of hippocampal neurogenesis, enhancement of neural plasticity, reduction of oxidative stress, suppression of inflammatory responses, and improvement of hypothalamic–pituitary–adrenal (HPA) axis dysfunction (10). At present, a multitude of animal studies have explored the therapeutic benefits of BBR on depression-related behaviors in rodent models; however, conflicting results have been reported in some experiments. For instance, Mengnan Huang et al. (16) reported that BBR exerted antidepressant effects by upregulating dopamine (DA) levels, whereas the study by Wen-Huang Peng et al. (17) found no significant regulatory effect of BBR on DA levels. Similarly, in a behavioral study, Ji-duo Shen et al. (18) found that BBR did not significantly affect the number of crossings or rearings in the open field test, which contradicts the findings of most other studies reporting behavioral efficacy.

Despite the findings from current animal research indicating that berberine possesses the potential for alleviating depression, the specific pharmacological mechanisms underlying its effects continue to be a subject of debate. Notably, no meta-analysis based on preclinical studies has yet been conducted to comprehensively evaluate the effects of berberine in depressive disorders. Consequently, this research intends to perform a comprehensive systematic review and meta-analysis by synthesizing pertinent animal studies to clarify the possible mechanisms through which berberine operates in DD. Furthermore, it seeks to offer a systematic scientific foundation for forthcoming clinical research.

## 2 Methods

This systematic review and meta-analysis were designed and conducted in accordance with the guidelines set forth by the Preferred Reporting Items for Systematic Reviews and Meta-Analyses (PRISMA) (19). The protocol was registered in the INPLASY database under the registration number INPLASY202560002.

### 2.1 Search strategy

In order to acquire an extensive understanding of preclinical research examining the impact of BBR on DD, we systematically searched five databases: PubMed, Embase, Web of Science, Cochrane Library, and OVID. The search was conducted up to March 20, 2025. Search strategies were discussed among the contributing authors to minimize the risk of missing eligible studies. The search strategy was developed based on the PICOS framework and involved combining terms related to the disease and intervention. In PubMed, Medical Subject Headings (MeSH) were employed, with search terms including”Berberine”“Coptis chinensis “ and “Depression”“Depressive Disorder”. The comprehensive search methodology is detailed in **Table 1**.

**Table 1:** Pubmed Search format.

Search	PUBMED
#1	(((((Berberine[MeSH Terms]) OR (Berberine Alkaloids[MeSH Terms])) OR (Coptis chinensis[MeSH Terms])) OR (Coptis[MeSH Terms])) OR (Coptidis rhizoma extract[MeSH Terms])) OR ((((((((((((((((((((((Berberine) OR (Berberine Alkaloids)) OR (Coptis chinensis)) OR (Coptis)) OR (Coptidis rhizoma extract)) OR (Coptidis rhizoma extract)) OR (Coptidis rhizoma extract)) OR (Berbines)) OR (Chinese Goldthread)) OR (Chinese Goldthreads)) OR (Goldthread, Chinese)) OR (Huang-Lian)) OR (Huang Lian)) OR (Copti)) OR (Goldthread)) OR (Goldthreads)) OR (Coptis teeta)) OR (Coptis teeta)) OR (Coptis chinensis root)) OR (huang lian extract)) OR (Rhizoma coptidis)) OR (huang-lian extract))
#2	((Depression[MeSH Terms]) OR (Depressive Disorder[MeSH Terms])) OR ((((((((((((((((((((((((((((((((Depression) OR (Depressive Disorder)) OR (Depressive Symptoms)) OR (Depressive Symptom)) OR (Symptom, Depressive)) OR (Emotional Depression)) OR (Depression, Emotional)) OR (Depressive Disorders)) OR (Disorder, Depressive)) OR (Disorders, Depressive)) OR (Neurosis, Depressive)) OR (Depressive Neuroses)) OR (Depressive Neurosis)) OR (Neuroses, Depressive)) OR (Depression, Endogenous)) OR (Depressions, Endogenous)) OR (Endogenous Depression)) OR (Endogenous Depressions)) OR (Melancholia)) OR (Melancholias)) OR (Unipolar Depression)) OR (Depression, Unipolar)) OR (Depressions, Unipolar)) OR (Unipolar Depressions)) OR (Depressive Syndrome)) OR (Depressive Syndromes)) OR (Syndrome, Depressive)) OR (Syndromes, Depressive)) OR (Depression, Neurotic)) OR (Depressions, Neurotic)) OR (Neurotic Depression)) OR (Neurotic Depressions))
#3	#1 AND #2

### 2.2 Inclusion criteria

The criteria for inclusion based on the PICOS framework were defined as follows: (1) Population: Experimental subjects were animal models with depression-like behaviors induced using recognized methods. Species (e.g., rats, mice), strain, sex, age, initial body weight, and sample size were not restricted. (2) Intervention: The experimental group received BBR monotherapy (via oral gavage, intraperitoneal injection, or other administration routes). The dose, frequency, and duration of BBR treatment were not restricted. (3) Comparison: The control group received either vehicle-treated control or served as a model control. The fundamental modeling method was consistent between groups. (4) Outcomes: Primary outcomes included improvement in depression-like behavioral indicators. Secondary outcomes could include changes in related neurobiochemical markers. (5) Study type:Published randomized controlled trials (RCTs). No language restrictions.

### 2.3 Exclusion criteria

1. Reviews, case reports, clinical studies, and *in vitro* experiments;
2. Preclinical studies unrelated to DD;
3. Duplicate publications;
4. Studies lacking experimental data;
5. Studies with a significant risk of bias in experimental outcomes.

### 2.4 Data extraction

The following data were independently extracted by two reviewers:

1. First author and year of publication;
2. Animal species, sex, weight range, and sample size;
3. Induction method of the DD animal model;
4. Intervention type, duration, and dosage in both model and treatment groups;
5. Outcome measures:weight, Number of crossing, total moving distance, Number of rearings, time duration of center square, Forced swimming test (FST), Tail suspension test (TST), Sucrose preference test (SPT), Novelty suppressed feeding test (NSFT), Brain-Derived Neurotrophic Factor (BDNF), Dopamine (DA), Serotonin (5-HT), Norepinephrine (NE), tumor necrosis factor α (TNF-α), interleukin-1β (IL-1β), interleukin 6 (IL-6). For data presented in graphical form, we first attempted to obtain the raw data by contacting the original authors. If raw data were unavailable, data were extracted using a digital data extraction tool. In instances where the data is presented as the standard error of the mean (SEM), the standard deviation (SD) can be determined utilizing the following formula: SD = SEM × √n (20).

### 2.5 Quality assessment

Two reviewers conducted an independent evaluation of the methodological quality of the studies included in the analysis of BBR as a treatment for DD, utilizing the 10-item SYRCLE’s risk of bias assessment tool (21). The assessment domains included: sequence generation, baseline characteristics, allocation concealment, random housing of animals, blinding of caregivers and investigators, random outcome assessment, blinding of outcome assessors, incomplete outcome data, selective outcome reporting, and other sources of bias. Any disagreements were resolved through consultation with the corresponding author.

### 2.6 Data analysis

Statistical evaluations were conducted utilizing STATA version 15.0 alongside Review Manager version 5.4. Given that the outcomes pertained to continuous variables, the results were articulated in terms of standardized mean differences (SMDs) accompanied by 95% confidence intervals (CIs). A p-value of less than 0.05 was deemed statistically significant. Heterogeneity was assessed using the I² statistic. A random-effects model was used to pool effect sizes. Subgroup analyses were performed to explore potential sources of heterogeneity. Prespecified subgroups included: animal species, sex, treatment duration (≤7 days, 8 – 14 days, ≥15 days), and dosage (≤20 mg/kg/day, 21 – 100 mg/kg/day, >100 mg/kg/day). When ≥10 studies were included, funnel plots were employed to evaluate the possibility of publication bias.

## 3 Results

### 3.1 Included studies

**Figure 1:**
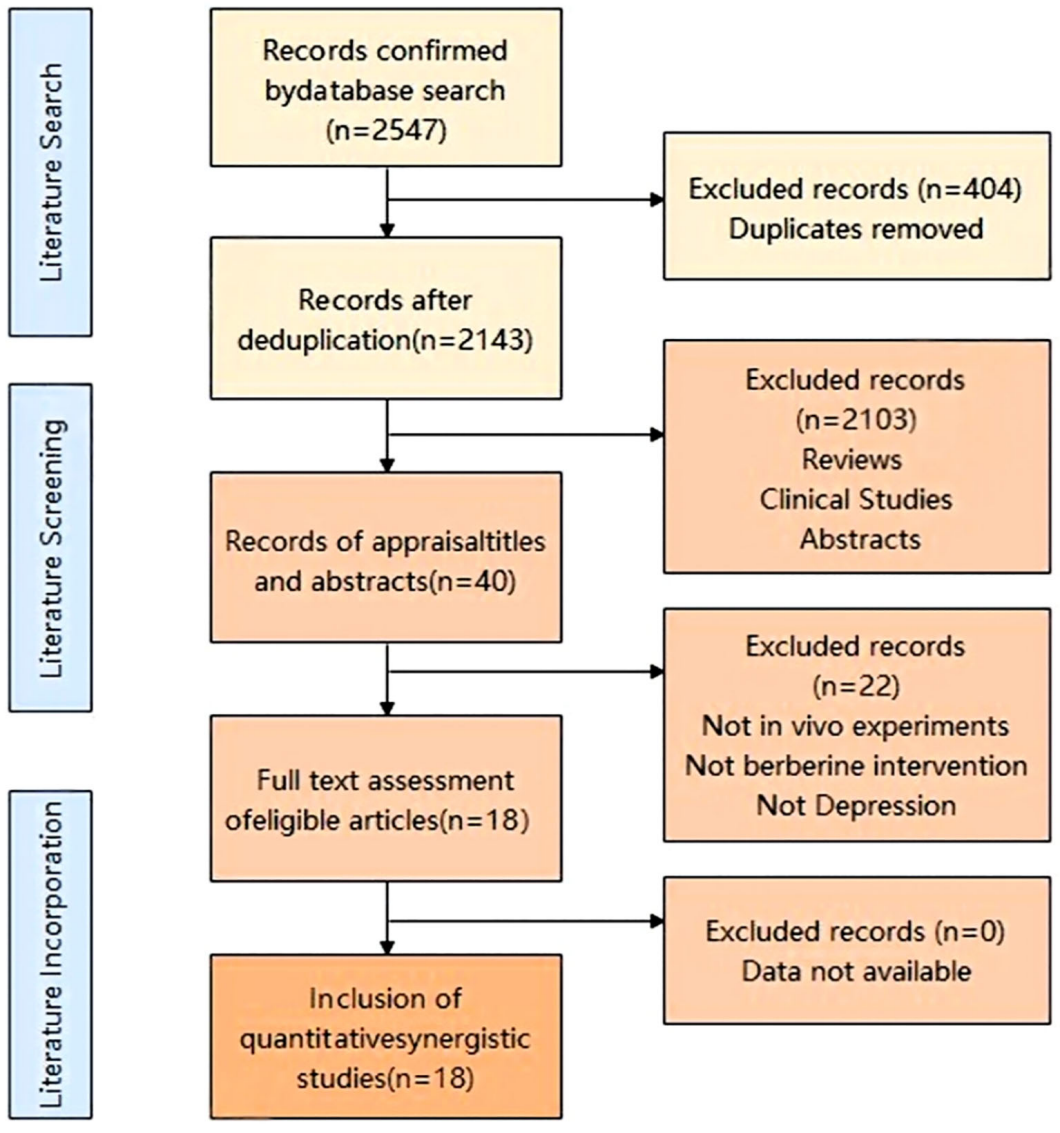
Flow diagram of the selection process.

A total of 2,547 potentially relevant studies were identified through database searches, including 169 from PubMed, 463 from Web of Science, 425 from Embase, 12 from the Cochrane Library, and 1,478 from OVID. After removing duplicates, 2,143 records were retained. Subsequent to the screening of both the title and abstract, a total of 2,103 records were eliminated due to their failure to satisfy the established inclusion criteria. After full-text evaluation, a total of 18 studies were incorporated into the conclusive analysis. ( (16–18, 22–36)). The procedure for selecting studies is depicted in **Figure 1**.

### 3.2 Characteristics of the included studies

A total of 18 English-language studies were included, comprising 338 animals, with 169 in the experimental groups and 169 in the model groups. Animal species included ICR mice (18, 22, 29, 36), C57BL/6 mice (26, 32, 34), C57/BL6J mice (27, 31, 33), C57BL/6N mice (35), Albino mice (24, 25), ICR albino mice (17), Wistar rats (16, 28) and Sprague-Dawley rats (23, 30). One study used only female animals (22). while seventeen studies used only male animals (16–18, 23–36). The experimental groups were treated with BBR, while the model groups received either a vehicle or blank control. In order to assess the impact of BBR on DD, the included studies were systematically summarized. Nine studies reported body weight changes, four studies reported the number of crossings, thirteen studies reported results from the FST, eleven studies used the SPT, and eight studies included the TST. Additional outcomes included: four studies using the NSFT; six studies reporting BDNF levels; four studies reporting DA levels; six studies measuring 5-HT levels; four studies reporting NE levels; five studies measuring TNF-α; four studies assessing IL - 1β; and three studies reporting IL - 6. Furthermore, eight studies reported total moving distance, five reported the number of rearings, and three assessed the duration of time spent in the center square. **Table 2** provides a comprehensive overview of the specific attributes of the studies incorporated in this analysis.

**Table 2:** Basic characteristics of the included studies.

Study(year)	Species (sex,n = BBR/modelgroup, weight)	Model method	BBR group (administration, drugdose, duration)	Model group (administration, drugdose, duration)	Outcome index
Jie Fan et al. (2017) (22)	ICR mice (Female, 10/10)	the bilateral ovarian resection (9 weeks)	By intraperitoneal, 10 mg/kg/d, 7 days	By intraperitoneal, normal saline, 7 days	②④⑥⑩
Wen-Huang Peng et al. (2007) (17)	ICR albino mice (Male, 10/10, 25 g)	By forced Swim Test and Tail Suspension Test (6-min)	By orally, 20 mg/kg/d, 60 minute	By orally, 0.9% saline water 10 ml/kg, 60 minute	②⑥⑦⑪ ⑫ ⑬
Ji-duo Shen et al. (2016) (18)	ICR mice (Male, 10/10, 18–22 g)	Corticosterone-induced depressive (21 days)	By subcutaneously, Corticosterone 40 mg/kg/d+By orally, Bbr 50 mg/kg/d, 21 days	By subcutaneously, Corticosterone 40 mg/kg/d, 21 days	①②④⑥⑧⑩
Bombi Lee et al. (2012) (23)	Sprague-Dawley rats (Male, 6/6, 260∼280g)	Morphine withdrawal from depression (10 days)	By subcutaneously, morphine 10~50mg/kg/d+By intraperitoneal 20mg/kg, 10 days	By subcutaneously, morphine 10~50mg/kg/d, 10 days	⑥
Shrinivas Kulkarni et al. (2008) (24)	Albino mice (Laca strain) (Male, 6/6, 22~30mg)	By forced Swim Test and Tail Suspension Test (6-min)	By intraperitoneal, 5 mg/kg/d, 30 minute	By intraperitoneal, normal saline, 30 minute	⑥⑦
Shrinivas Kulkarni et al. (2007) (25)	Albino mice (Laca strain) (Male, 6/6, 22~30mg)	By forced Swim Test and Tail Suspension Test (6-min)	By intraperitoneal, 5mg/kg/d, 30 minute	By intraperitoneal, normal saline, 30 minute	⑥⑦
Mengnan Huang et al. (2023) (16)	Wistar rats (Male, 10/10, 170 ± 10g)	CUMS pressure source consisting of six different types of pressure sources (35 days)	By intragastrically, 50mg/kg/d, 14 days	By intragastrically, normal saline, 1 ml/100 g/d, 14 days	①③④⑧⑩⑪ ⑫ ⑬
Qin Gong et al. (2019) (26)	C57BL/6 mice (Male, 8/8, 18~20g)	Corticosterone-induced depressive (21 days)	By subcutaneously, Corticosterone 20 mg/kg/d,21 days+By orally, Bbr 150 mg/kg/d, 14 days	By subcutaneously, Corticosterone 20 mg/kg/d,21 days	①⑧⑩
Zhifang Deng et al. (2018) (27)	C57/BL6J mice (Male, 8/8)	Chronic social defeat stress (10 days)	By Chronic social defeat stress+By orally, Bbr 100 mg/kg/d, 10 days	By Chronic social defeat stress, 10 days	①③⑤⑥⑦⑧⑩
Qiang-Song Wang et al. (2020) (28)	Wistar rats (Male, 8/8, 180~220g)	Chronic stress (35 days)	By orally, 5mg/kg/d, 3 days	By orally, normal saline 10ul/g/d, 3 days	①③④⑧⑪ ⑫ ⑬
Qi Wang et al. (2022) (29)	ICR mice (Male, 10/10, 22~24g)	Chronic unpredictable mild stress (21 days)	By orally, 25mg/kg/d, 21days	By orally, 0.9% saline, 3 days	①②③④⑥⑦⑧⑨⑫
Xiaohui Zhu et al. (2017) (30)	Sprague Dawley rats (Male, 10/10, 200~220g)	chronic stress depression model (28 days)	By orally, 200mg/kg/d, 28days	By orally, 0.9% saline, 2ml, 28 days	①⑥⑧
Lu Yang et al. (2023) (31)	C57BL/6J mice (Male, 9/9)	Chronic unpredictable mild stress procedure (28 days)	By gavage, 10mg/kg/d, 21days	By gavage, distilled water, 21 days	③⑥⑦⑧⑭ ⑮
Yueheng Tang et al. (2024) (32)	C57BL/6 mice (Male, 6/6,)	Chronic restraint stress (21 days)	By gavage, 300mg/kg/d, 21days	By gavage, 0.9% saline, 21 days	①③⑥⑦⑩⑫ ⑬ ⑭ ⑯
Li-tao Yi et al. (2021) (33)	C57BL/6J mice (Male, 12/12, 20~22 g)	Chronic stress mode (28 days)	By orally, 100mg/kg/d, 28 days	By orally, normal saline, 28 days	⑧⑨
Ping-Yuan Ge et al. (2023) (34)	C57BL/6 mice (Male, 10/10, 18~22 g)	Chronic unpredictable mild stress (28 days)	By gavage, 10mg/kg/d, 7 days	By gavage, 0.5% CMC-Na, 7 days	①③⑤⑥⑨⑪ ⑫ ⑭ ⑮
Zongshi Qin et al. (2023) (35)	C57BL/6N mice (Male, 18/18)	Corticosterone-induced depressive (35 days)	By intragastrically, Corticosterone 10mg/kg/d, 35 days+Bbr 100mg/kg/d, 29 days	By intragastrically, Corticosterone 10mg/kg/d, 35 days	③⑤⑥⑦⑧⑭ ⑮ ⑯
Ya-Min Liu et al. (2017) (36)	ICR mice (Male, 12/12, 22 ± 2g)	Chronic unpredictable mild stress (28 days)	By orally, 100mg/kg/d, 28 days	By orally, 0.9% saline, 28 days	⑧⑨⑭ ⑮ ⑯

①weight, ②Number of crossing, ③total distance, ④Number of rearings, ⑤time duration of center square, ⑥Forced swimming test, ⑦Tail suspension test, ⑧Sucrose preference test, ⑨Novelty suppressed feeding test, ⑩ BDNF, ⑪ DA, ⑫ 5-HT, ⑬ NE, ⑭ TNF-α, ⑮ IL-1β, ⑯ IL-6.

### 3.3 Quality of the included studies

Quality assessment of the included studies revealed that 17 out of 18 reported using randomization for animal allocation, while one study did not clearly specify the use of randomization. Only three studies provided baseline characteristics for the groups, and the remaining 14 did not clarify whether such characteristics were considered. No study reported on allocation concealment between groups. As all studies were conducted under identical laboratory conditions, animal housing was considered randomized. None of the studies explicitly mentioned blinding of personnel. Three studies employed randomized outcome assessment, and three described blinded outcome assessment. A total of six studies provided comprehensive outcome data. There was no indication of selective reporting or any other forms of bias detected. An overview of the methodological quality of the studies included can be found in **Table 3**.

**Table 3:** The methodological quality of included studies.

Study	Year	A	B	C	D	E	F	G	H	J	K	Total
Jie Fan (22)	2017	+	+	?	+	?	+	+	?	+	+	7
Wen-Huang Peng (17)	2007	?	+	–	+	?	+	+	–	+	+	6
Ji-duo Shen (18)	2016	?	?	?	+	?	?	?	–	+	+	3
Bombi Lee (23)	2012	?	?	?	+	?	?	+	+	+	+	5
Shrinivas Kulkarni (24)	2008	?	?	?	+	?	?	?	–	+	+	3
Shrinivas Kulkarni (25)	2007	?	?	?	+	?	?	?	?	+	+	3
Mengnan Huang (16)	2023	?	?	?	+	?	?	?	–	+	+	3
Qin Gong (26)	2019	?	?	?	+	?	?	?	–	+	+	3
Zhifang Deng (27)	2018	?	?	?	+	?	?	?	+	+	+	4
Qiang-Song Wang (28)	2020	?	?	?	+	?	?	?	–	+	+	3
Qi Wang (29)	2022	?	?	?	+	?	?	?	+	+	+	4
XiaoHui Zhu (30)	2017	?	?	?	+	?	?	?	?	+	+	3
Lu Yang (31)	2023	?	+	?	+	?	+	?	+	+	+	6
Yueheng Tang (32)	2024	?	?	?	+	?	?	?	+	+	+	4
Li-tao Yi (33)	2021	?	?	?	+	?	?	?	+	+	+	4
Ping-Yuan Ge (34)	2023	?	?	?	+	?	?	?	–	+	+	3
Zongshi Qin (35)	2023	?	?	?	+	?	?	?	?	+	+	3
Ya-Min Liu (36)	2017	?	?	?	+	?	?	?	–	+	+	3

A: Sequence generation; B: Baseline characteristics; C: Allocation concealment; D: Random housing; E: Blinding for trial researchers and caregivers; F: Random outcome assessment; G: Blinding for outcome assessors; H: Incomplete outcome data; I: Selective outcome reporting; J: Other sources of bias. +: low-risk of bias;?: unclear-risk of bias; -, high-risk of bias.

### 3.4 Results of the meta-analysis

#### 3.4.1 Body weight change

**Figure 2:**
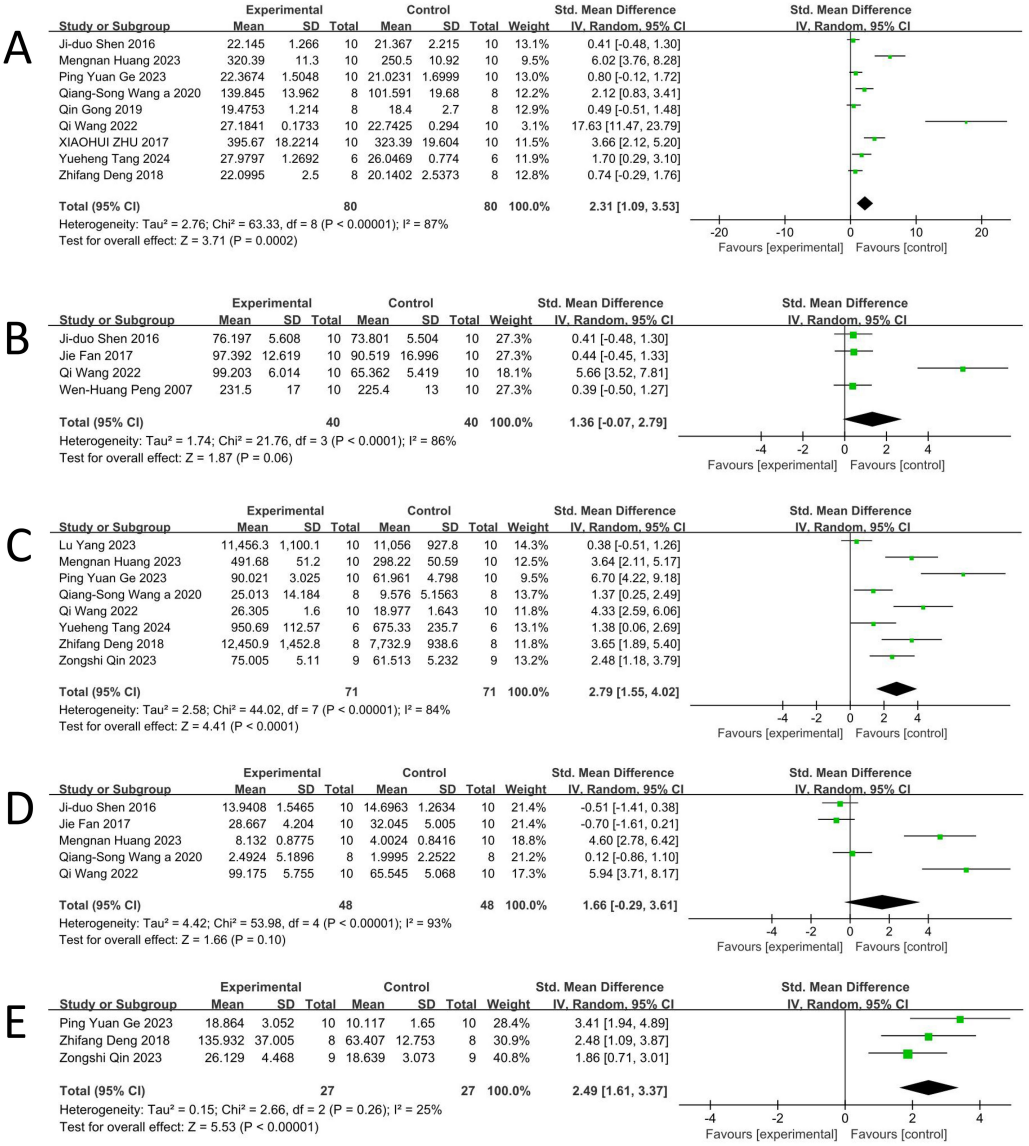
Forest plot: effect of berberine on the **(A)** weight, **(B)** Number of crossing, **(C)**Total distance, **(D)** Number of rearings, **(E)** Time duration of center square.

According to data from nine studies (16, 18, 26–30, 32, 34), a random-effects meta-analysis indicated that BBR significantly increased BWC levels in DD animal models [n = 160, SMD = 2.31, 95% CI (1.09, 3.53), p < 0.0002; **Figure 2A**]. Substantial heterogeneity was observed among the included studies (I² = 87%, p < 0.00001; **Figure 2A**).

#### 3.4.2 Behavioral experiment

##### 3.4.2.1 Number of crossing

According to data from four studies (17, 18, 22, 29), the random-effects meta-analysis showed that there was no statistically significant difference in the number of crossings between the BBR-treated group and the control group [n = 80, SMD = 1.36, 95% CI (−0.07, 2.79), p = 0.06; **Figure 2B**]. Considerable heterogeneity was observed among the included studies (I² = 86%, p < 0.0001; **Figure 2B**).

##### 3.4.2.2 Total distance

According to data from eight studies (16, 27–29, 31, 32, 34, 35), a random-effects meta-analysis demonstrated that BBR significantly increased the total distance traveled in DD animal models [n = 142, SMD = 2.79, 95% CI (1.55, 4.02), p < 0.0001; **Figure 2C**]. Considerable heterogeneity was observed among the included studies (I² = 84%, p < 0.00001; **Figure 2C**).

##### 3.4.2.3 Number of rearings

According to data from five studies (16, 18, 22, 28, 29), a random-effects meta-analysis revealed that there was no statistically significant difference between the BBR-treated group and the control group in the number of rearings [n = 96, SMD = 1.66, 95% CI (–0.29, 3.61), p = 0.10; **Figure 2D**]. Considerable heterogeneity was observed among the included studies (I² = 93%, p < 0.00001; **Figure 2D**).

##### 3.4.2.4 Time duration of center square

According to data from three studies (27, 34, 35), a random-effects meta-analysis indicated that BBR significantly increased the time spent in the center square in DD animal models [n = 54, SMD = 2.49, 95% CI (1.61, 3.37), p < 0.00001; **Figure 2E**]. Only mild heterogeneity was detected among the included studies (I² = 25%, p = 0.26; **Figure 2E**).

##### 3.4.2.5 FST

**Figure 3:**
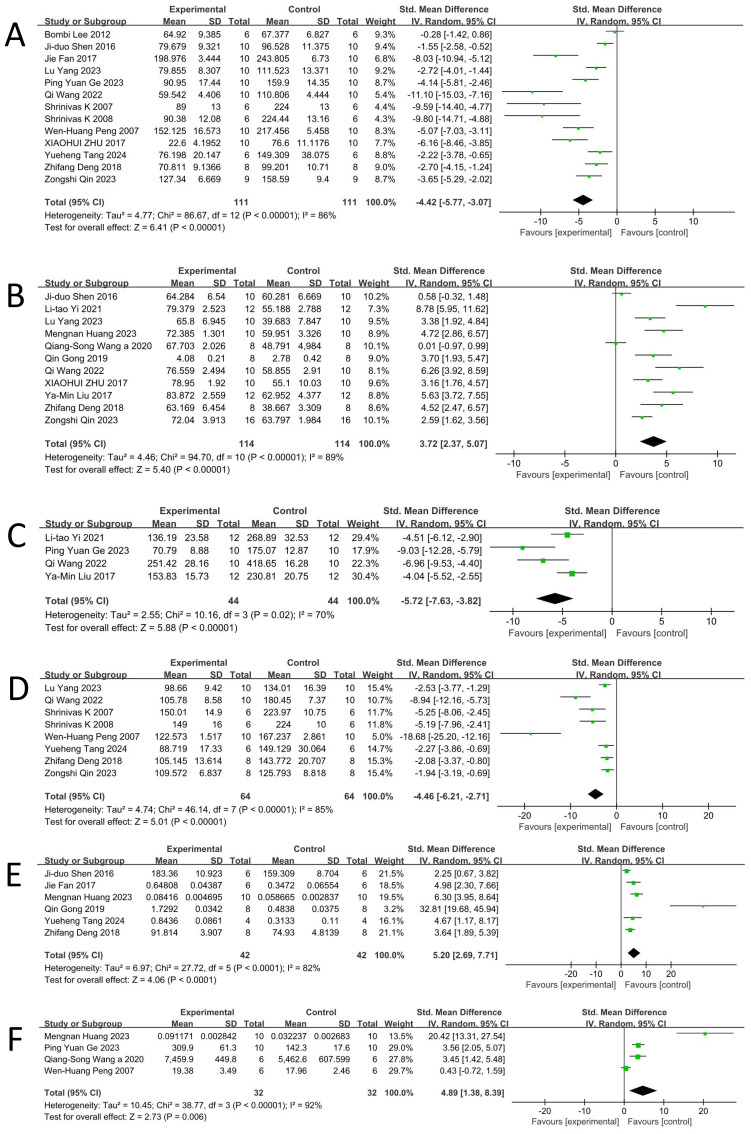
Forest plot: effect of berberine on the **(A)** Forced swimming test, **(B)** Sucrose preference test, **(C)** Novelty suppressed feeding test, **(D)** Tail suspension test **(E)** BDNF, **(F)** DA.

According to data from 13 studies (17, 18, 22–25, 27, 29–32, 34, 35), a random-effects meta-analysis revealed that BBR significantly reduced immobility time in the FST in DD animal models [n = 222, SMD = -4.42, 95% CI (-5.77, -3.07), p < 0.00001; **Figure 3A**]. Considerable heterogeneity was observed across the included studies (I² = 86%, p < 0.00001; **Figure 3A**).

##### 3.4.2.6 SPT

According to data from 11 studies (16, 18, 26–31, 33, 35, 36), a random-effects meta-analysis demonstrated that BBR significantly increased sucrose preference in DD animal models [n = 228, SMD = 3.72, 95% CI (2.37, 5.07), p < 0.00001; **Figure 3B**]. Substantial heterogeneity was detected among the included studies (I² = 89%, p < 0.00001; **Figure 3B**).

##### 3.4.2.7 NSFT

According to data from four studies (29, 33, 34, 36), a random-effects meta-analysis indicated that BBR significantly reduced latency to feed in the NSFT in DD animal models [n = 88, SMD = -5.72, 95% CI (-7.63, -3.82), p < 0.00001; **Figure 3C**]. Significant heterogeneity was observed among the included studies (I² = 70%, p = 0.02; **Figure 3C**).

##### 3.4.2.8 TST

Based on data from eight studies (17, 24, 25, 27, 29, 31, 32, 35), a random-effects meta-analysis demonstrated that BBR significantly reduced immobility time in the Tail Suspension Test (TST) in animal models of DD [n = 128, SMD = -4.46, 95% CI (-6.21, -2.71), p < 0.00001; **Figure 3C**]. Considerable heterogeneity was detected across studies (I² = 85%, p < 0.00001; **Figure 3D**).

#### 3.4.3 BDNF

Based on data from six studies (16, 18, 22, 26, 27, 32), a random-effects meta-analysis revealed that BBR significantly increased BDNF levels in animal models of DD [n = 84, SMD = 5.20, 95% CI (2.69, 7.71), p < 0.0001; **Figure 3D**]. Considerable heterogeneity was observed among the included studies (I² = 82%, p < 0.0001; **Figure 3E**).

#### 3.4.4 Neurotransmitter-related indicators

##### 3.4.4.1 DA

Based on data from four studies (16, 17, 28, 34), a random-effects meta-analysis showed that BBR significantly increased DA levels in animal models of DD [n = 64, SMD = 4.89, 95% CI (1.38, 8.39), p = 0.006; **Figure 3E**]. Considerable heterogeneity was observed among the included studies (I² = 92%, p < 0.00001; **Figure 3F**).

##### 3.4.4.2 5-HT

**Figure 4:**
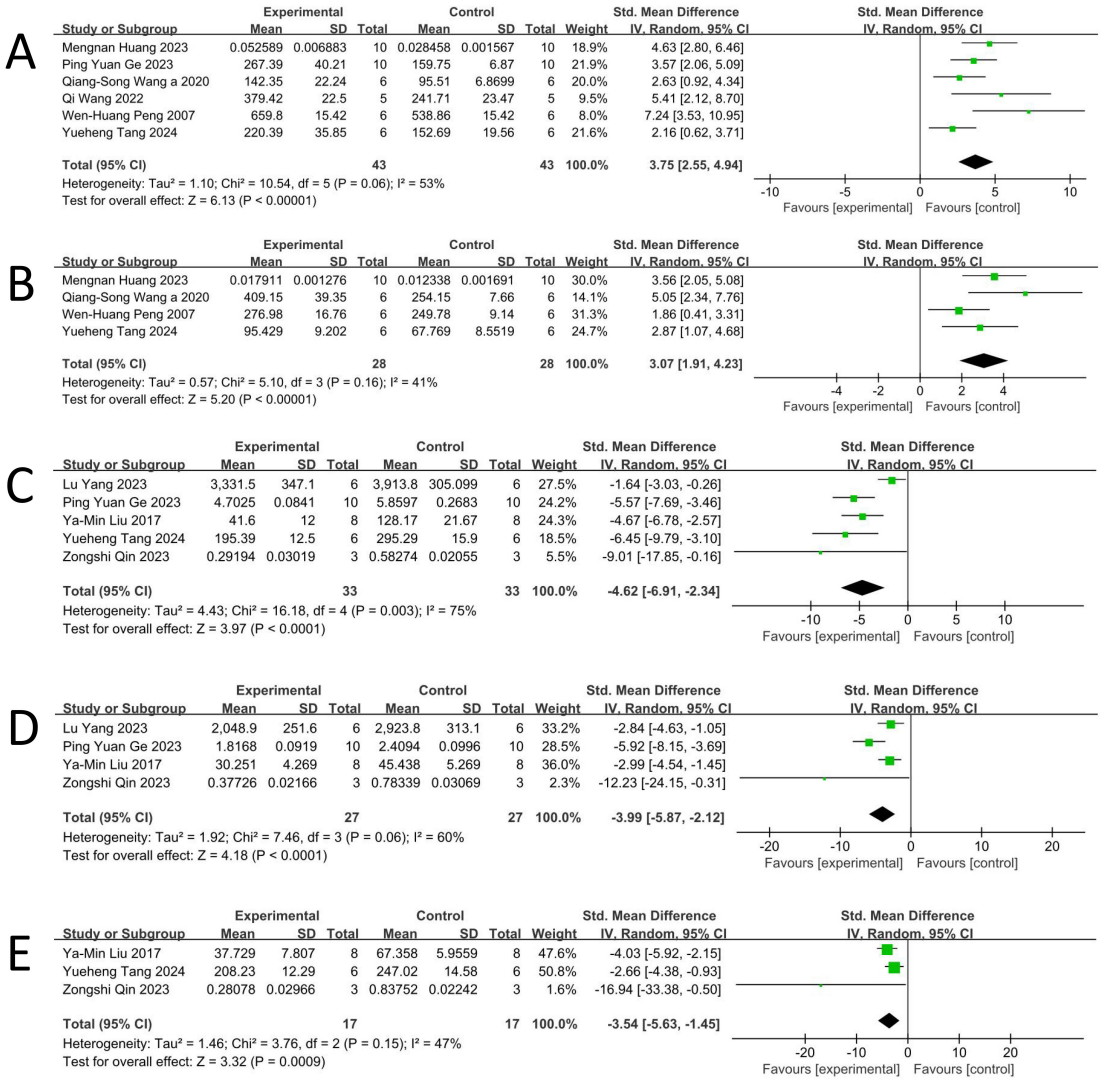
Forest plot: effect of berberine on the **(A)** 5-HT, **(B)** NE, **(C)** TNF-α, **(D)** IL-1β, **(E)** IL-6.

Based on data from six studies (16, 17, 28, 29, 32, 34), a random-effects meta-analysis demonstrated that BBR significantly increased 5-HT levels in animal models of DD [n = 86, SMD = 3.75, 95% CI (2.55, 4.94), p < 0.00001; **Figure 3E**]. A moderate level of heterogeneity was observed (I² = 53%, p = 0.06; **Figure 4A**).

##### 3.4.4.3 NE

Based on data from four studies (16, 17, 28, 32), a random-effects meta-analysis demonstrated that BBR significantly increased NE levels in animal models of DD [n = 56, SMD = 3.07, 95% CI (1.91, 4.23), p < 0.00001; **Figure 4B**]. A moderate level of heterogeneity was observed (I² = 41%, p = 0.16; **Figure 4B**).

#### 3.4.5 Inflammation-related indicators

##### 3.4.5.1 TNF-α

Based on data from five studies (31, 32, 34–36), a random-effects meta-analysis demonstrated that BBR significantly reduced TNF-α levels in animal models of DD [n = 66, SMD = -4.62, 95% CI (-6.91, -2.34), p < 0.0001; **Figure 4C**]. Considerable heterogeneity was observed among the included studies (I² = 75%, p = 0.003; **Figure 4C**).

##### 3.4.5.2 IL - 1β

Based on data from four studies (31, 34–36), a random-effects meta-analysis demonstrated that BBR significantly reduced IL - 1β levels in animal models of DD [n = 54, SMD = -3.99, 95% CI (-5.87, -2.12), p < 0.0001; **Figure 4D**]. Significant heterogeneity was observed among the included studies (I² = 60%, p = 0.06; **Figure 4D**).

##### 3.4.5.3 IL - 6

Based on data from three studies (32, 35, 36), a random-effects meta-analysis demonstrated that BBR significantly reduced IL - 6 levels in animal models of DD [n = 34, SMD = -3.54, 95% CI (-5.63, -1.45), p = 0.0009; **Figure 4E**]. A moderate level of heterogeneity was observed among the included studies (I² = 47%, p = 0.15; **Figure 4E**).

### 3.5 Subgroup analysis

Due to the substantial heterogeneity among studies, subgroup analyses were conducted based on animal species, sex, treatment duration, and treatment dosage for 16 outcomes, including weight, number of crossings, total distance, number of rearings, time spent in the center square, FST, TST, SPT, NSFT, BDNF, DA, 5-HT, NE, TNF-α,IL-1β, and IL - 6. The results indicated that sex and treatment duration may contribute to heterogeneity in FST; treatment duration may contribute to heterogeneity in SPT; both treatment duration and dosage may contribute to heterogeneity in NSFT; animal species, treatment duration, and dosage may contribute to heterogeneity in DA; treatment duration may contribute to heterogeneity in IL - 1β; dosage may contribute to heterogeneity in total distance; and sex and treatment duration may contribute to heterogeneity in the number of rearings. Significant heterogeneity remained across studies. Detailed results are presented in **Supplementary Tables 1–16**.

### 3.6 Publication bias

**Figure 5:**
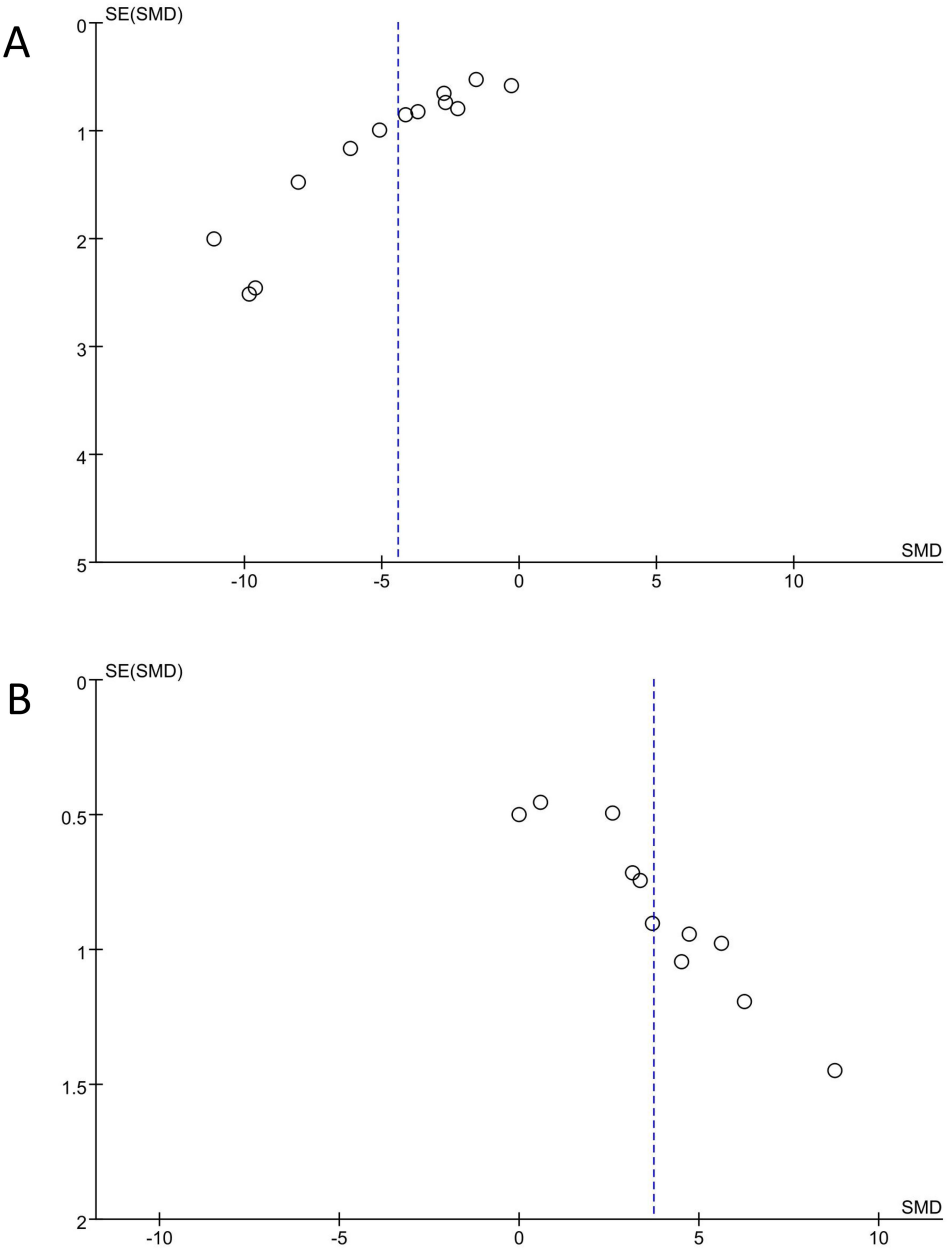
Results of trim and fill method. **(A)** Forced swimming test, **(B)** Sucrose preference test.

Funnel plots were employed to evaluate the possibility of publication bias in both the FST and SPT analyses, and publication bias was detected, as shown in **Figures 5A, B**.

## 4 Discussion

### 4.1 Summary of evidence

As far as we are aware, this represents the inaugural preclinical systematic review and meta-analysis that assesses the pharmacological impacts and possible mechanisms of action of BBR in the management of DD.A total of 18 studies involving 338 experimental animals were included. Subgroup analyses were performed for all outcome measures. The results suggested that sex and treatment duration may contribute to heterogeneity in the FST; treatment duration in the SPT; treatment duration and dosage in the NSFT; animal species, treatment duration, and dosage in DA; treatment duration in IL - 1β; dosage in total distance; and sex and treatment duration in the number of rearings. Publication bias was assessed for two outcomes with sample sizes greater than 10 (FST and SPT), and evidence of publication bias was detected. Overall, the findings indicated that BBR significantly ameliorated depressive-like behaviors in DD animal models, increased body weight, BDNF, DA, 5-HT, and NE levels, and decreased TNF-α, IL - 1β, and IL - 6 levels.

### 4.2 Possible protective mechanisms of BBR

This systematic review and meta-analysis show that BBR treatment increased body weight in mice with depressive-like behavior (16, 18, 26–30, 32, 34). Previous studies (26), have shown that BBR alleviates depressive-like behavior and indirectly promotes weight gain by restoring mitochondrial oxidative phosphorylation function suppressed by corticosterone (CORT), thereby improving energy supply. In addition, BBR exerts anti-inflammatory effects by reducing pro-inflammatory cytokines such as IL - 1β and TNF-α, and increasing anti-inflammatory cytokines such as IL - 4, thereby improving intestinal inflammation and nutrient absorption, which contributes to weight recovery (37). Furthermore, BBR may promote weight gain by repairing chronic stress-induced gastrointestinal mucosal damage (e.g., gastric mucosa, atrophy of ileal/colonic microvilli) and reducing inflammatory infiltration, thereby restoring nutrient absorption (30).

This systematic review and meta-analysis show that BBR ameliorated depressive-like behaviors in mice, as evidenced by increased total distance traveled and time spent in the center area in the open field test, reduced immobility time in the forced swimming test and tail suspension test, increased sucrose intake in the sucrose preference test, and decreased latency to feed in the novelty suppressed feeding test (16–18, 22–36). However, BBR did not significantly alter the number of crossings or rearings in the open field test (16–18, 22, 28, 29).

Several studies have confirmed that BBR significantly improves depressive-like behaviors in mice through multiple synergistic pathways: by inhibiting monoamine oxidase A (MAO-A) activity, thereby increasing 5-HT and NE levels in the prefrontal cortex and hippocampus (17), directly reducing immobility in the forced swimming and tail suspension tests and enhancing sucrose preference; suppressing hyperactivation of the hypothalamic-pituitary-adrenal (HPA) axis by lowering serum corticosterone (18), and acting via 5-HT_2_ receptor-mediated upregulation of hippocampal BDNF expression, promotion of cAMP response element-binding protein (CREB) phosphorylation, and inhibition of eukaryotic elongation factor 2 phosphorylation (22), thereby enhancing synaptic plasticity, increasing exploratory behavior in the open field test, and shortening latency in the novelty suppressed feeding test. These effects are accompanied by increased neuronal activity in the prefrontal cortex and hippocampus, further reinforcing behavioral improvement.

This systematic review and meta-analysis show that BBR improved depressive-like behaviors in mice by increasing NE, 5-HT, and DA levels in the hippocampus and prefrontal cortex (16, 17, 28, 29, 32, 34). Reduced monoamine neurotransmitter levels are associated with the development of depression (38). Studies have shown that BBR reduces the degradation of monoamine neurotransmitters (NE, 5-HT, DA) in the hippocampus and prefrontal cortex by inhibiting monoamine oxidase A (MAO-A) activity and enhances dopamine biosynthesis by upregulating dopa decarboxylase (DDC) expression (29). BBR promotes the conversion of tryptophan to 5-HT by activating tryptophan hydroxylase 1 and inhibits indoleamine 2,3-dioxygenase 1 activity, blocking pathological shunting of tryptophan to kynurenine, thereby elevating central 5-HT levels (34). Furthermore, BBR activates the PI3K/AKT/CREB/BDNF signaling pathway, suppresses neuroinflammation and apoptosis, enhances neuronal survival and synaptic plasticity, and promotes the release of NE, 5-HT, and DA (32). Notably, BBR may indirectly enhance the synthesis and release of monoamine neurotransmitters (e.g., NE, 5-HT, DA) by modulating gut microbiota composition and its metabolic products, such as short-chain fatty acids (16).

This systematic review and meta-analysis show that BBR improved depressive-like behaviors in mice by enhancing BDNF levels (16, 18, 22, 26, 27, 32). BDNF is a type of neurotrophic factor that enhances the differentiation of neural stem cells into neurons. It also facilitates the survival, proliferation, and maturation of neurons specifically within the adult olfactory bulb and the dentate gyrus regions (39), and has been validated as a key factor promoting synaptic plasticity to exert antidepressant effects (18). BBR indirectly promotes neurogenesis by regulating BDNF levels. BBR reduces depressive-like behaviors by increasing BDNF expression in the hippocampal CA1 region, as evidenced by improvements in sucrose preference test, FST, and open field test(OFT) performance (18), Overexpression of BDNF can reverse the effects of miR-34b-5p and miR-470-5p on depressive-like behaviors in chronic unpredictable mild stress mice (40). In addition, BBR improves depression through the BDNF/CREB/eukaryotic elongation factor 2 pathway, with an onset of action 2 – 4 weeks faster than selective serotonin reuptake inhibitors (22).

This systematic review and meta-analysis show that BBR suppressed inflammatory responses by reducing IL - 1β, IL - 6, and TNF-α levels. Inflammatory responses are closely associated with depressive symptoms (31, 32, 34–36). Pro-inflammatory cytokines have the capacity to diminish the availability of 5-HT, DA, and NE by enhancing both the expression and activity of presynaptic 5-HT reuptake transporters. Additionally, they activate indoleamine 2,3-dioxygenase (IDO), which subsequently leads to a reduction in the synthesis of monoamine precursors (41). Inflammation also influences the levels of growth factors, including BDNF, within the dentate gyrus of the hippocampus, which results in compromised neuronal integrity (42). Numerous studies have shown that the antidepressant effects of BBR are associated with its anti-inflammatory properties. BBR reduces IL - 1β, IL - 6, and TNF-α levels in the hippocampus and inhibits microglial activation, thereby alleviating depressive symptoms induced by CUMS (36). In addition, BBR alleviates depressive symptoms and reduces hippocampal neuronal dysfunction in CUMS mice by enhancing the binding of Trim65 to NOD-like receptor family pyrin domain containing 3 (NLRP3) and promoting NLRP3 ubiquitination, thereby inhibiting NLRP3 inflammasome activation (31).

## 5 Limitations and recommendations in methodology

Although the preclinical application of BBR in DD is promising, several limitations should be considered. This systematic review identified publication bias, which may be partly related to the quality of the included studies. Although some studies reported the use of randomization, none provided details on the randomization methods. The majority of outcomes presented in this systematic review demonstrated considerable heterogeneity. Although certain studies showed a decrease in heterogeneity after conducting predetermined subgroup analyses, the majority did not investigate the factors contributing to this heterogeneity. Furthermore, the results of this systematic review revealed a lack of toxicological information, as none of the studies incorporated within the review provided thorough assessments of toxicology. Moreover, the limitations of animal models and the uncertainty in clinical translation warrant careful consideration. Although animal models are indispensable for mechanistic exploration and preliminary efficacy assessment, they cannot fully replicate key aspects of human disease, such as the complex pathophysiological environment, pharmacokinetic profiles, and immune responses. Consequently, the existence of species differences implies that the efficacy and safety of BBR observed in animal models within this systematic review may deviate from outcomes in future human clinical trials. It is recommended to establish standardized and comprehensive experimental protocols. Researchers should report negative results transparently to promote broader acceptance. Harmonizing outcome measurement and calculation methods, as well as presenting original data, may also improve the accuracy of future evaluations and reduce bias. Additionally, it is recommended that standardized experimental models be adopted, incorporating multiple dose-gradient groups (e.g., ≤20 mg/kg/d, 21 – 100 mg/kg/d, >100 mg/kg/d) to allow for a more comprehensive assessment of BBR’s dose-response relationship and safety profile.

## 6 Conclusion

In conclusion, our meta-analysis and systematic review demonstrated that BBR significantly ameliorated depressive symptoms by increasing body weight, BDNF, DA, 5-HT, and NE levels, and reducing TNF-α, IL - 1β, and IL - 6 levels, thereby improving depressive-like behaviors. The antidepressant mechanisms of BBR may be closely associated with the regulation of neurotransmitter levels, reduction of oxidative stress, and suppression of inflammatory responses. Nevertheless, considering the variability and the methodological rigor of the studies included in this analysis, it is essential to interpret these results cautiously. Subsequent research employing more stringent experimental frameworks and thorough methodologies is required to confirm the protective benefits of BBR in DD.

## Data Availability

The raw data supporting the conclusions of this article will be made available by the authors, without undue reservation.
