# Supplementary material for: The effects of berberine on depressive symptoms: a systematic review and meta-analysis of preclinical studies
Source: Front Psychiatry. 2025 Dec 29;16:1653929. doi: 10.3389/fpsyt.2025.1653929 (PMC12794032; doi:10.3389/fpsyt.2025.1653929)
Supplement: Supplementary file 1 [file Supplementaryfile1.docx]

**Supplementary Table 1** The subgroup analyses of Weight

| **Parameter** | **Subgroup** |  | **No. of studies** | **SMD [95% CI]** | **I**2 **(%)** | **P for heterogeneity** | **Test for subgroup difference** |
| --- | --- | --- | --- | --- | --- | --- | --- |
| Weight | species | mice | 6 | 3.76  [1.73, 5.78] | 78 | P=0.0003 | I^2^=74.2% p=0.05 |
|  |  | RAT | 3 | 1.38  [0.14, 2.61] | 84 | P=0.03 |  |
|  | treatment cycles（days） | ≤7days | 2 | 1.38 [0.10,2.66] | 62 | P=0.03 | I^2^=21.4% p=0.28 |
|  |  | 8-14days | 3 | 2.15 [-0.22,4.51] | 90 | P=0.08 |  |
|  |  | ≥15days | 4 | 3.95  [1.01, 6.89] | 92 | P <0.0001 |  |
|  | treatment dose(mg/kg) | ≤20mg/kg/d | 2 | 1.38  [0.10, 2.66] | 62 | P=0.1 | I^2^=34.9% p=0.22 |
|  |  | 21-100mg/kg/d | 4 | 4.42 [1.27,7.58] | 94 | P<0.00001 |  |
|  |  | >100mg/kg/d | 3 | 1.87  [0.07, 3.68] | 83 | P=0.03 |  |

**Supplementary Table 2** The subgroup analyses of Number of crossing

| **Parameter** | **Subgroup** |  | **No.of studies** | **SMD[95% CI]** | **I**^2^ **(%)** | **P  for heterogeneity** | **Test for subgroup  difference** |
| --- | --- | --- | --- | --- | --- | --- | --- |
| Number of  crossing | sex | Male | 3 | 1.87 [0.30.4.04] | 91 | P=0.09 | I^2^=29.8% p=0.23 |
|  |  | Female | 1 | 0.44  [-0.45, 1.33] |  | P=0.33 |  |
|  | treatment cycles（days） | ≤7days | 2 | 0.41  [-0.22, 1.04] | 0 | P=0.20 | I^2^=0% p=0.34 |
|  |  | ≥15days | 2 | 2.94  [-2.20, 8.08] | 95 | P=0.26 |  |
|  | treatment dose(mg/kg) | ≤20mg/kg/d | 2 | 0.41  [-0.22, 1.04] | 0 | P=0.20 | I^2^=0% p=0.34 |
|  |  | 21-100mg/kg/d | 2 | 2.94  [-2.20, 8.08] | 95 | P=0.26 |  |

**Supplementary Table 3** The subgroup analyses of Forced swimming test

| **Parameter** | **Subgroup** |  | **No.of studies** | **SMD[95% CI]** | **I**^2^ **(%)** | **P  for heterogeneity** | **Test for subgroup  difference** |
| --- | --- | --- | --- | --- | --- | --- | --- |
| Forced swimming test | species | mice | 10 | 4.25  [-5.57, -2.92] | 81 | P <0.00001 | I^2^=0% p=0.71 |
|  |  | RAT | 2 | -3.13  [-8.89, 2.63] | 95 | P=0.29 |  |
|  | sex | Male | 12 | 4.10 [-5.43,-2.77] | 85 | P <0.00001 | I^2^=82.8% p=0.02 |
|  |  | Female | 1 | -8.03[-10.94, -5.12] | 86 | P <0.00001 |  |
|  | treatment cycles（days） | ≤7days | 5 | -6.55 [-8.67,-4.43] | 65 | P <0.00001 | I^2^=89% p=0.007 |
|  |  | 8-14days | 2 | -1.44 [-3.81, 0.93] | 85 | P=0.23 |  |
|  |  | ≥15days | 6 | -3.94 [-5.66,-2.21] | 85 | P <0.00001 |  |
|  | treatment dose(mg/kg) | ≤20mg/kg/d | 7 | -5.02 [-7.30,-2.74] | 89 | P <0.0001 | I^2^=0% p=0.81 |
|  |  | 21-100mg/kg/d | 4 | -3.99 [-6.32,-1.67] | 87 | P =0.0007 |  |
|  |  | >100mg/kg/d | 2 | 4.09 [-7.95,-0.23] | 87 | P =0.04 |  |

**Supplementary Table 4** The subgroup analyses of Sucrose preference test

| **Parameter** | **Subgroup** |  | **No.of studies** | **SMD[95% CI]** | **I2 (%)** | **P  for heterogeneity** | **Test for subgroup  difference** |
| --- | --- | --- | --- | --- | --- | --- | --- |
| Sucrose preference test | species | mice | 8 | 4.20  [2.58, 5.82] | 89 | P <0.00001 | I^2^=0% p=0.32 |
|  |  | RAT | 3 | 2.55  [-0.30,5.40] | 92 | P <0.00001 |  |
|  | treatment cycles（days） | ≤7days | 1 | 0.01  [-0.97,0.99] | 89 | P=0.99 | I^2^=93.8%  P <0.00001 |
|  |  | 8-14days | 2 | 4.05  [2.71, 5.39] | 0 | P <0.00001 |  |
|  |  | ≥15days | 8 | 4.14  [2.57, 5.71] | 89 | P <0.00001 |  |
|  | treatment dose(mg/kg) | ≤20mg/kg/d | 2 | 1.65  [-1.66,4.95] | 93 | P=0.33 | I^2^=14.3% p=0.31 |
|  |  | 21-100mg/kg/d | 7 | 4.52  [2.58, 6.47] | 91 | P <0.00001 |  |
|  |  | >100mg/kg/d | 2 | 3.37  [2.27, 4.47] | 0 | P <0.00001 |  |

**Supplementary Table 5** The subgroup analyses of Tail suspension test

| **Parameter** | **Subgroup** |  | **No.of studies** | **SMD[95% CI]** | **I**^2^ **(%)** | **P  for heterogeneity** | **Test for subgroup  difference** |
| --- | --- | --- | --- | --- | --- | --- | --- |
| Tail suspension test | treatment cycles（days） | ≤7days | 3 | -8.78 [-14.49,-3.07] | 87 | P=0.03 | I^2^=60.3% p=0.08 |
|  |  | 8-14days | 1 | -2.08  [-3.37, -0.80] |  | P=0.01 |  |
|  |  | ≥15days | 4 | -2.33  [-2.98, -1.67] | 0 | P <0.00001 |  |
|  | treatment dose(mg/kg) | ≤20mg/kg/d | 4 | -6.69 [-10.69,-2.70] | 88 | P=0.001 | I^2^=57.7% p=0.09 |
|  |  | 21-100mg/kg/d | 3 | -2.19  [-2.92, -1.46] | 0 | P <0.00001 |  |
|  |  | >100mg/kg/d | 1 | -2.27 [-3.86,-0.69] |  | P=0.005 |  |

**Supplementary Table 6** The subgroup analyses of Novelty suppressed feeding test

| **Parameter** | **Subgroup** |  | **No.of studies** | **SMD[95% CI]** | **I**^2^ **(%)** | **P  for heterogeneity** | **Test for subgroup  difference** |
| --- | --- | --- | --- | --- | --- | --- | --- |
| Novelty suppressed feeding test | treatment cycles（days） | ≤7days | 1 | -9.03 [-12.28,-5.79] |  | P <0.00001 | I^2^=81.2% p=0.02 |
|  |  | ≥15days | 3 | -4.86  [-6.30, -3.41] | 48 | P <0.00001 |  |
|  | treatment dose(mg/kg) | ≤20mg/kg/d | 1 | -9.03 [-12.28,-5.79] | 48 | P <0.00001 | I^2^=81.2% p=0.02 |
|  |  | 21-100mg/kg/d | 3 | -4.86  [-6.30, -3.41] |  | P <0.00001 |  |

**Supplementary Table 7** The subgroup analyses of BDNF

| **Parameter** | **Subgroup** |  | **No.of studies** | **SMD[95% CI]** | **I**^2^ **(%)** | **P  for heterogeneity** | **Test for subgroup  difference** |
| --- | --- | --- | --- | --- | --- | --- | --- |
| BDNF | species | mice | 5 | 5.02  [2.10, 7.93] | 83 | P=0.0007 | I^2^=0% p=0.50 |
|  |  | RAT | 1 | 6.30  [3.95, 8.64] |  | P <0.00001 |  |
|  | sex | Male | 5 | 5.44  [2.39, 8.49] | 85 | P=0.0005 | I^2^=0% p=0.82 |
|  |  | Female | 1 | 4.98  [2.30, 7.66] |  | P=0.0003 |  |
|  | treatment cycles（days） | ≤7days | 1 | 4.98  [2.30, 7.66] |  | P=0.0003 | I^2^=50.5% p=0.13 |
|  |  | 8-14days | 3 | 8.86  [2.94, 14.78] | 90 | P=0.003 |  |
|  |  | ≥15days | 2 | 2.93  [0.80, 5.07] | 35 | P=0.007 |  |
|  | treatment dose(mg/kg) | ≤20mg/kg/d | 1 | 4.98  [2.30, 7.66] |  | P=0.0003 | I^2^=0% p=0.52 |
|  |  | 21-100mg/kg/d | 3 | 3.91  [1.77, 6.05] | 75 | P=0.0003 |  |
|  |  | >100mg/kg/d | 1 | 18.00  [-9.54, 45.54] | 94 | P=0.20 |  |

**Supplementary Table 8** The subgroup analyses of DA

| **Parameter** | **Subgroup** |  | **No.of studies** | **SMD[95% CI]** | **I**^2^ **(%)** | **P  for heterogeneity** | **Test for subgroup  difference** |
| --- | --- | --- | --- | --- | --- | --- | --- |
| DA | species | mice | 3 | 2.40  [0.14, 4.66] | 85 | P=0.04 | I^2^=95.5%  P <0.00001 |
|  |  | RAT | 1 | 20.42  [13.31, 27.54] |  | P <0.00001 |  |
|  | treatment cycles（days） | ≤7days | 3 | 2.40  [0.14, 4.66] | 85 | P=0.04 | I^2^=95.5%  P <0.00001 |
|  |  | 8-14days | 1 | 20.42  [13.31, 27.54] |  | P <0.00001 |  |
|  | treatment dose(mg/kg) | ≤20mg/kg/d | 3 | 2.40  [0.14, 4.66] | 85 | P=0.04 | I^2^=95.5%  P <0.00001 |
|  |  | 21-100mg/kg/d | 1 | 20.42  [13.31, 27.54] |  | P <0.00001 |  |

**Supplementary Table 9** The subgroup analyses of 5-HT

| **Parameter** | **Subgroup** |  | **No.of studies** | **SMD[95% CI]** | **I**^2^ **(%)** | **P  for heterogeneity** | **Test for subgroup  difference** |
| --- | --- | --- | --- | --- | --- | --- | --- |
| 5-HT | species | mice | 4 | 4.01  [2.18,5.85] | 61 | P <0.0001 | I^2^=0% P=0.76 |
|  |  | RAT | 2 | 3.60  [1.64, 5.56] | 39 | P=0.003 |  |
|  | treatment cycles（days） | ≤7days | 3 | 3.86  [1.97, 5.74] | 49.9 | P <0.0001 | I^2^=0% P=0.76 |
|  |  | 8-14days | 1 | 4.63  [2.80, 6.46] | 18.9 | P <0.00001 |  |
|  |  | ≥15days | 2 | 3.45  [0.34, 6.56] | 31.1 | P=0.03 |  |
|  | treatment dose(mg/kg) | ≤20mg/kg/d | 3 | 3.86  [1.97, 5.74] | 49.9 | P <0.0001 | I^2^=64.2% P=0.06 |
|  |  | 21-100mg/kg/d | 2 | 4.81  [3.22, 6.41] | 28.5 | P <0.00001 |  |
|  |  | >100mg/kg/d | 1 | 2.16  [0.62, 3.71] | 21.6 | P=0.006 |  |

**Supplementary Table 10** The subgroup analyses of NE

| **Parameter** | **Subgroup** |  | **No.of studies** | **SMD[95% CI]** | **I**^2^ **(%)** | **P  for heterogeneity** | **Test for subgroup  difference** |
| --- | --- | --- | --- | --- | --- | --- | --- |
| NE | species | mice | 2 | 2.26  [1.13, 3.39] | 0 | P <0.0001 | I^2^=71.4% P=0.06 |
|  |  | RAT | 2 | 3.92  [2.59, 5.24] | 0 | P <0.00001 |  |
|  | treatment cycles（days） | ≤7days | 2 | 3.24  [0.14, 6.33] | 76 | P=0.04 | I^2^=0% P=0.85 |
|  |  | 8-14days | 1 | 3.56  [2.05, 5.08] |  | P <0.0001 |  |
|  |  | ≥15days | 1 | 2.87  [1.07, 4.68] |  | P=0.002 |  |
|  | treatment dose(mg/kg) | ≤20mg/kg/d | 2 | 3.24  [0.14, 6.33] | 76 | P=0.04 | I^2^=0% P=0.85 |
|  |  | 21-100mg/kg/d | 1 | 3.56  [2.05, 5.08] |  | P <0.00001 |  |
|  |  | >100mg/kg/d | 1 | 2.87  [1.07, 4.68] |  | P=0.002 |  |

**Supplementary Table 11** The subgroup analyses of TNF-α

| **Parameter** | **Subgroup** |  | **No.of studies** | **SMD[95% CI]** | **I**^2^ **(%)** | **P  for heterogeneity** | **Test for subgroup  difference** |
| --- | --- | --- | --- | --- | --- | --- | --- |
| TNF-α | treatment cycles（days） | ≤7days | 1 | -5.57 [-7.69,-3.46] | 75 | P <0.00001 | I^2^=0% P=0.5 |
|  |  | ≥15days | 4 | -4.37 [-7.13,-1.62] |  | P=0.002 |  |
|  | treatment dose(mg/kg) | ≤20mg/kg/d | 2 | -3.52  [-7.37, 0.32] | 89 | P=0.07 | I^2^=0% P=0.25 |
|  |  | 21-100mg/kg/d | 2 | -4.91 [-6.95,-2.86] | 0 | P <0.00001 |  |
|  |  | >100mg/kg/d | 1 | -6.45  [-9.79, -3.10] |  | P=0.0002 |  |

**Supplementary Table 12** The subgroup analyses of IL-1β

| **Parameter** | **Subgroup** |  | **No.of studies** | **SMD[95% CI]** | **I**^2^ **(%)** | **P  for heterogeneity** | **Test for subgroup  difference** | |
| --- | --- | --- | --- | --- | --- | --- | --- | --- |
| IL-1β | treatment cycles（days） | ≤7days | 1 | -5.92 [-8.15,-3.69] |  | P <0.00001 | | I^2^=78.7% P=0.03 |
|  |  | ≥15days | 3 | -5.92 [-8.15,-3.69] | 14 | P <0.00001 | |  |
|  | treatment dose(mg/kg) | ≤20mg/kg/d | 2 | -4.31 [-7.32,-1.29] | 78 | P=0.005 | | I^2^=0% P=0.76 |
|  |  | 21-100mg/kg/d | 2 | -5.65  [-13.83,2.54] | 56 | P=0.18 | |  |

**Supplementary Table 13** The subgroup analyses of IL-6

| **Parameter** | **Subgroup** |  | **No.**  **of studies** | **SMD[95% CI]** | **I**^2^ **(%)** | **P  for heterogeneity** | **Test for subgroup  difference** |
| --- | --- | --- | --- | --- | --- | --- | --- |
| IL-6 | treatment dose(mg/kg) | 21-100mg/kg/d | 2 | -7.80  [-19.30,3.70] | 57 | P=0.18 | I^2^=0% P=0.39 |
|  |  | >100mg/kg/d | 1 | -2.66 [-4.38,-0.93] |  | P=0.002 |  |

**Supplementary Table 14** The subgroup analyses of total distance

| **Parameter** | **Subgroup** |  | **No.of studies** | **SMD[95% CI]** | **I**^2^ **(%)** | **P  for heterogeneity** | **Test for subgroup  difference** |
| --- | --- | --- | --- | --- | --- | --- | --- |
| total distance | species | mice | 6 | 2.96  [1.32, 4.59] | 87 | P=0.0004 | I^2^=0% P=0.17 |
|  |  | RAT | 2 | 2.44  [0.22, 4.67] | 82 | P=0.03 |  |
|  | treatment cycles（days） | ≤7days | 2 | 3.92  [-1.31,9.14] | 93 | P=0.14 | I^2^=26% P=0.26 |
|  |  | 8-14days | 2 | 3.64  [2.49, 4.80] | 0 | P <0.00001 |  |
|  |  | ≥15days | 4 | 2.03  [0.46, 3.61] | 84 | P=0.01 |  |
|  | treatment dose(mg/kg) | ≤20mg/kg/d | 3 | 2.50  [-0.04, 5.03] | 91 | P=0.05 | I^2^=70.9% P=0.03 |
|  |  | 21-100mg/kg/d | 3 | 3.85  [2.89, 4.81] | 0 | P <0.00001 |  |
|  |  | >100mg/kg/d | 2 | 1.93  [0.85, 3.02] | 27 | P=0.0005 |  |

**Supplementary Table 15** The subgroup analyses of Number of rearings

| **Parameter** | **Subgroup** |  | **No.of studies** | **SMD[95% CI]** | **I**^2^ **(%)** | **P  for heterogeneity** | **Test for subgroup  difference** |
| --- | --- | --- | --- | --- | --- | --- | --- |
| Number of rearings | species | mice | 3 | 1.31  [-1.34, 3.95] | 93 | P <0.00001 | I^2^=0% P=0.71 |
|  |  | RAT | 2 | 2.29 [-2.10,6.68] | 94 | P <0.0001 |  |
|  | sex | Male | 4 | 2.36  [-0.24, 4.96] | 94 | P=0.13 | I^2^=79% P=0.03 |
|  |  | Female | 1 | -0.70 [-1.61,0.21] |  | P=0.07 |  |
|  | treatment cycles（days） | ≤7days | 2 | -0.31 [-1.11,0.49] | 30 | P=0.44 | I^2^=91.7%  P <0.00001 |
|  |  | 8-14days | 1 | 4.60  [2.78, 6.42] | 0 | P <0.00001 |  |
|  |  | ≥15days | 2 | 2.63  [-3.69, 8.95] | 96 | P=0.41 |  |
|  | treatment dose(mg/kg) | ≤20mg/kg/d | 2 | -0.31  [-1.11, 0.49] | 30 | P=0.23 | I^2^=59.7% P=0.12 |
|  |  | 21-100mg/kg/d | 3 | 3.26  [-1.11, 7.63] | 96 | P=0.14 |  |

**Supplementary Table 16** The subgroup analyses of time duration of center square

| **Parameter** | **Subgroup** |  | **No.of studies** | **SMD[95% CI]** | **I**^2^ **(%)** | **P  for heterogeneity** | **Test for subgroup  difference** |
| --- | --- | --- | --- | --- | --- | --- | --- |
| time duration of center square | treatment cycles（days） | ≤7days | 1 | 3.41  [1.94, 4.89] |  | P <0.00001 | I^2^=24.8% P=0.26 |
|  |  | 8-14days | 1 | 2.48  [1.09, 3.87] |  | P=0.0005 |  |
|  |  | ≥15days | 1 | 1.86  [0.71, 3.01] |  | P=0.002 |  |
|  | treatment dose(mg/kg) | ≤20mg/kg/d | 1 | 3.41  [1.94, 4.89] |  | P <0.00001 | I^2^=54.8% P=0.14 |
|  |  | 21-100mg/kg/d | 2 | 2.11  [1.22, 3.00] | 0 | P <0.00001 |  |
